# The Solid-State Structures of Dimethylzinc and Diethylzinc

**DOI:** 10.1002/anie.201105099

**Published:** 2011-09-14

**Authors:** John Bacsa, Felix Hanke, Sarah Hindley, Rajesh Odedra, George R Darling, Anthony C Jones, Alexander Steiner

**Affiliations:** Department of Chemistry, University of LiverpoolLiverpool L69 7ZD (UK) E-mail: a.steiner@liv.ac.uk; Surface Science Research Centre, University of LiverpoolLiverpool L69 3BX (UK); SAFC HitechPower Road, Bromborough, Wirral, CH62 3QF (UK)

**Keywords:** density functional calculations, organometallic compounds, solid-state structures, X-ray diffraction, zinc

The synthesis of dimethylzinc (Me_2_Zn) and diethylzinc (Et_2_Zn) by Frankland in the mid-nineteenth century marks a cornerstone in the history of chemistry.^1^ Not only were they among the first organometallic compounds, but studies on their chemical reactions and vapor densities led to the first clear exposition of valency theory.^2^ Since then both compounds have found widespread applications: They are important reagents in organic synthesis,^3^ for example in the enantioselective alkylation of carbonyls^4^ and imines^5^ and in cyclopropanation reactions.^6^ Their high vapor pressures have led to extensive uses in metalorganic chemical vapor deposition (MOCVD) for the preparation of wide band gap II–VI semiconducting films (e.g. ZnS, ZnSe, ZnTe),^7^ ZnO nanostructures, and as *p*-dopant precursors for III–V semiconductors (e.g. GaAs, InP, Al_*x*_Ga_1−*x*_As), which have numerous electronic and photonic applications.^8^

Despite their prominence in chemical and materials synthesis, the solid-state structures of these prototypical organometallic systems remained elusive.^9^ Me_2_Zn and Et_2_Zn feature the smallest and lightest molecules containing metal atoms in the condensed phase. At standard conditions they exist as volatile, pyrophoric liquids (Me_2_Zn: m.p. −42 °C, b.p. 46 °C; Et_2_Zn: m.p. −28 °C, b.p. 118 °C), which are moderately soft Lewis acids that form adducts with a variety of ligands.^10^ Structural studies of the gas phase have revealed a linear coordination mode.^11^ Solid-state structures of diaryl^12^ and bulky dialkylzinc derivatives^13^ exhibit monomeric structures with the exception of Ph_2_Zn, which forms a loosely bonded dimer.12a Here we present a combined crystallographic and computational study, which shows that Me_2_Zn and Et_2_Zn exhibit a rich solid-phase behavior. We have used density functional theory with dispersion corrections including an uncertainty analysis for the van der Waals forces^14^ to elucidate the detailed structure of the crystals.

Single crystals were grown from samples sealed in quartz capillaries. Me_2_Zn was found to exist in two polymorphic forms, which are enantiotropic undergoing a reversible transition at 180 K (Figure 1). We were able to determine X-ray crystal structures of both the high-temperature phase, α-Me_2_Zn, and the low-temperature phase, β-Me_2_Zn.^15^ A single crystal of α-Me_2_Zn was grown by gradually cooling the sample to 200 K. X-ray data indicate a tetragonal unit cell with Laue symmetry 4/*mmm*. Refinement in space group *P*4_2_/*mnm* exhibits two molecules per unit cell aligned orthogonally to each other and parallel to the *ab* plane occupying *D*_2*h*_ sites. This setting, however, is incompatible with the molecular three-fold symmetry. Also, the C atom exhibits a high anisotropic displacement in the z direction. Refinement in the monoclinic space group *P*2_1_/*n* as a pseudo-merohedral twin decreases the site symmetry to *C_i_* and tilts the molecule by 5.6° with respect to the *ab* plane (Figure 1). As a result, the two methyl groups adopt a staggered conformation. The C—Zn—C unit is strictly linear and the Zn—C bond measures 1.927(6) Å in good agreement with the gas-phase structure.^11^ Each zinc atom is surrounded by four methyl groups of neighboring molecules with two shorter and two longer CH_3_⋅⋅⋅Zn distances (C⋅⋅⋅Zn 3.491(7) and 3.708(7) Å, respectively). The twinning can be attributed to a two-dimensional disorder as stacks of molecules along the *c* axis can adopt two tilt orientations (see the Supporting Information). The disorder leads to an entropic free-energy term of *k*_B_ *T* ln2 per stack, contributing to the stabilization of the α phase at higher temperature.

**Figure 1:**
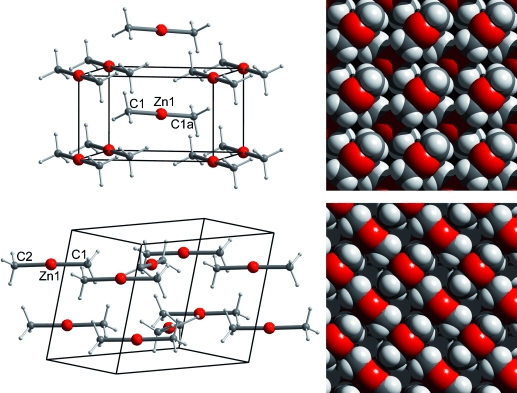
X-ray crystal structures of α-Me_2_Zn (top) and β-Me_2_Zn (bottom). Ball-and-stick models are viewed along and space-filling models perpendicular to the *ab* planes. Zinc atoms are shown in red.

Our calculated structure is in good general agreement with the X-ray structure^15^ and we obtain a cohesive energy of 0.61(4) eV per molecule, of which 94 % is due to van der Waals forces. A molecular tilt with respect to the *ab* plane is also favored, albeit by a larger angle of 9.8°. The molecules have strictly linear C—Zn—C units and the Zn—C bonds compare favorably (1.933 Å), as do the intermolecular Zn—C distances of 3.357 and 3.718 Å.

Cooling Me_2_Zn below 180 K leads to the formation of polycrystalline β-Me_2_Zn. A single crystal of this phase was obtained by annealing the sample at 150 K. The β phase is more compact than the α phase and features a more complex but ordered structure (see the Supporting Information for a detailed structural comparison of α and β phases). β-Me_2_Zn exhibits a monoclinic unit cell of space group symmetry *P*2_1_/*n* containing four molecules. These are arranged in square grid-type layers parallel to the *ab* plane, with each Zn atom forming two T-shaped interactions with methyl groups of neighboring molecules showing Zn⋅⋅⋅C distances of 3.327(14) and 3.424(14) Å (Figure 1). These layers are stacked upon each other such that Me_2_Zn molecules of adjacent layers align in parallel fashion with a Zn⋅⋅⋅C distance of 3.455(15) Å. The molecules are virtually linear (C—Zn—C 178.2(6)°) with Zn—C bond lengths of 1.911(14) (Zn1—C1) and 1.920(13) Å (Zn1—C2). The low quality of the data, which were obtained from a sample containing several single-crystal domains, did not allow us to locate H positions accurately. However, careful analysis of the various conformers shows that one particular conformation exhibits appropriate intermolecular distances with the methyl groups arranged eclipsed to each other (see the Supporting Information).

Computationally, we find several distinct local minima for this phase that are broadly consistent with the Zn—C distances, but all have slightly higher cohesive energies than α-Me_2_Zn. A sensitivity analysis14d shows that these energies depend strongly on the radius of the Zn atoms and the ad hoc damping function in the dispersion correction.14c This slightly unphysical dependence of the binding energies and geometries on fitted parameters in the description of the van der Waals forces is responsible for the inability to describe this particular phase theoretically. Note, however, that we do very well in determining the structures of α-Me_2_Zn and Et_2_Zn, which could not be achieved with a selection of alternative methods.

Diethylzinc formed sufficiently large single crystals when the capillary was gradually cooled to 100 K. It exhibits a tetragonal body-centered unit cell of space group symmetry *I*4_1_*md* containing four molecules.^15^ These display *C*_2*v*_ symmetry; the zinc atom and the four carbon atoms are co-planar, the C—Zn—C angle is very slightly bent (176.2(4)°), while both ethyl groups (Zn—C—C 115.9(4)°) are in mutual *cis* position. The Zn—C bonds, measuring 1.948(5) Å, are marginally longer than in Me_2_Zn, as observed for the gas-phase structures.^11^ The molecules pack in a polar arrangement such that ethyl groups all face in one direction along *z*. Each molecule has eight neighbors to which it is aligned orthogonally (Figure 2). The immediate intermolecular environment of the Zn atom is occupied by two CH_2_ (Zn⋅⋅⋅C1 3.254(6) Å) and two CH_3_ groups of neighboring molecules (Zn⋅⋅⋅C2 3.504(6) Å). Our calculations predict that the cohesion of 0.88(6) eV per molecule is 99 % due to dispersion forces, and the calculated structure agrees well with the experimental data.^15^

**Figure 2:**
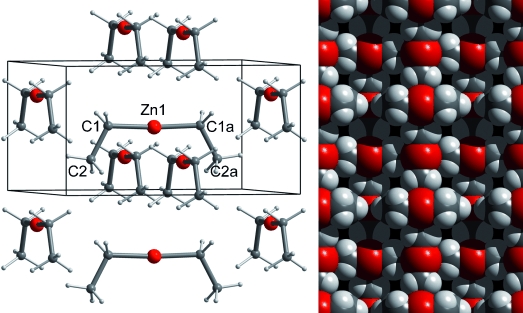
X-ray crystal structure of Et_2_Zn. The ball-and-stick model is viewed along and the space-filling model perpendicular to the *ab* plane. Zinc atoms are shown in red.

A qualitative way to map the intermolecular interactions are Hirshfeld surfaces,^16^ as presented in Figure 3 a for α-Me_2_Zn and Et_2_Zn (see also the Supporting Information). The red patches demonstrate relatively close contacts between the alkyl groups and the Zn atoms of neighboring molecules. To quantify the electronic contributions, we obtained the density of states (DOS, Figure 3 a). In the occupied states, we find a sharp peak because of the highest occupied molecular orbital (HOMO) of α-Me_2_Zn and Et_2_Zn, respectively. The HOMO of α-Me_2_Zn has three nodal planes along the molecular axis (Figure 3 b), such that the overlap between the terminal lobe of one molecule and the center of its neighbor is exactly zero and gives rise to the sharp feature in the DOS. For each crystal, there are two very broad peaks below the HOMO levels, suggesting band formation because of the intermolecular hybridization of the Zn—C bonding orbitals (around −3 eV) and a hybridization between Me and Et groups of adjacent molecules (around −5 eV and lower). For α-Me_2_Zn, the HOMO-1 has only two nodal planes along the molecular axis such that the broadening in the DOS arises from the overlap of the terminal lobe in one molecule with the central lobe of its neighbor. Finally, the relative orientation of the methyl groups is governed by the degenerate HOMO-2 and HOMO-3 levels, which allow for greater intermolecular overlap for the staggered conformation. Similar arguments explain the DOS for Et_2_Zn. Overall, our analysis shows that Me_2_Zn and Et_2_Zn crystals are held together largely by van der Waals forces, with a fine balance of orbital overlaps giving rise to small covalent interactions that determine the detailed crystal structure. Given the difficulties in describing all interactions at once with current density functionals (see the Supporting Information), our systems therefore present a particular challenge for the development of electronic structure methods which is only partially solved at present.

**Figure 3:**
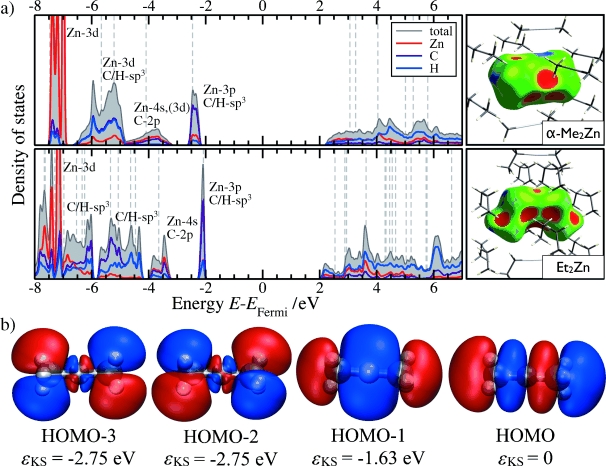
a) Left: Density of states for α-Me_2_Zn (top) and Et_2_Zn (bottom). The Fermi levels were aligned in the middle of each band gap for better comparison between the structures. The vertical dashed lines correspond to the free molecule energy eigenvalues. Right: Structure and Hirshfeld surfaces. The color denotes the distance from the nearest atom outside the surface. b) Four highest occupied Kohn–Sham orbitals of the free molecule and their energy difference to the HOMO.

In summary, we have determined the solid-state structures of Me_2_Zn and Et_2_Zn using X-ray crystallography and density functional theory. Me_2_Zn undergoes a reversible solid–solid phase transition at 180 K; its high-temperature phase shows a two-dimensional disorder. All three structures contain linear diorganozinc molecules, which interact through weak intermolecular interactions with small covalent contributions. To properly describe the interplay of all the relevant interactions and to obtain the correct crystal structure remains challenging for density functional methods. This demonstrates that the lower dialkylzinc systems continue to be very intriguing and still provide valuable data for ongoing scientific discussions more than 160 years after their discovery.

## Supplementary material

Detailed facts of importance to specialist readers are published as ”Supporting Information”. Such documents are peer-reviewed, but not copy-edited or typeset. They are made available as submitted by the authors.
