# Supplementary material for: The Solid-State Structures of Dimethylzinc and Diethylzinc
Source: Angew Chem Int Ed Engl. 2011 Sep 14;50(49):11685–7. doi: 10.1002/anie.201105099 (PMC3326375; doi:10.1002/anie.201105099)
Supplement: Supplementary file 1 [file anie0050-11685-SD1.pdf]

Angewandte Chemie
Eine Zeitschrift der Gesellschaft Deutscher Chemiker

Supporting Information
© Wiley-VCH 2011
69451 Weinheim, Germany

The Solid-State Structures of Dimethylzinc and Diethylzinc

*John Bacsa, Felix Hanke, Sarah Hindley, Rajesh Odedra, George R. Darling, Anthony C. Jones, and Alexander Steiner**

anie_201105099_sm_miscellaneous_information.pdf

Table of contents

1. Crystal data and structure refinement	S2
2. Structural comparison of α-Me_2Zn and β-Me_2Zn	S4
3. Hirshfeld surface analysis	S6
4. Two-dimensional disorder of α-Me_2Zn	S10
5. Assessment of the conformation of the methyl groups of β-Me_2Zn	S11
6. Details of Density Functional Calculations	S12
7. Atomic coordinates for all calculated crystal structures	S14

1. Crystal data and structure refinement

Samples were contained in quartz capillaries (0.5 mm bore diameter), which were flame sealed. The capillaries were mounted vertically on the X-ray goniometer and cooled with a cold stream of N_2. Crystals of α-Me_2Zn and Et_2Zn were grown from the melt by gradually cooling the samples. Crystals of β-Me_2Zn were obtained by moving a hot wire along the outside of the capillary. Crystal data were collected on a Bruker Apex diffractometer using MoK_{α} radiation (λ = 0.71073 Å). Crystal structures were refined with SHELX by full-matrix least squares against F^2 using all data.[S1] Note, that the coverage of reflection data was limited due to the experimental setup, which only enabled ω rotation of the vertically aligned capillary.

α-Me_2Zn: C_2H_6Zn, M_r = 95.44, T = 200 K, refinement in monoclinic space group $P2_1$/n as pseudo-merohedral twin, a = 6.856(5), b = 6.842(5), c = 4.182(3) Å, β = 89.970(11)°, V = 196.2(2) Å^3, Z = 2, ρ = 1.616 g cm^{-3}, $2\theta_{max}$ = 50°, 266 unique reflections (coverage 0.78), R_{int} = 0.0318, $R1$ ($I > 2\sigma(I)$) = 0.0197, $wR2$ (all data) = 0.0548. The Zn atom was refined anisotropically and the C-atom isotropically. The torsion angle of the methyl group was refined while its geometry was constrained. Alternative refinement in tetragonal spacegroup $P4_2$/mnm with split C-position: a = 6.849(4), c = 4.182(3), V = 196.2(2), Z = 2, ρ = 1.616 g cm^{-3}, $2\theta_{max}$ = 50°, 110 unique reflections (coverage 0.99), R_{int} = 0.0343, $R1$ ($I > 2\sigma(I)$) = 0.0149, $wR2$ (all data) = 0.0411. The Zn atom was refined anisotropically, the C-atom isotropically, while H-atoms were constrained to parent C-atoms.

β-Me_2Zn: C_2H_6Zn, M_r = 95.44, T = 150 K, monoclinic space group $P2_1$/n, a = 7.457(13), b = 7.497(14), c = 7.484(13) Å, β = 114.12(3) °, V = 381.9(12) Å^3, Z = 4, ρ = 1.660 g cm^{-3}, $2\theta_{max}$ = 50°, 460 unique reflections (coverage 0.69), R_{int} = 0.0721, $R1$ ($I > 2\sigma(I)$) = 0.0566, $wR2$ (all data) = 0.1427. Non-H atoms were refined anisotropically. The methyl groups were constrained to parent C-atoms. The orientation of the methyl groups was obtained from Hirshfeld surface analysis (see Figure S6).

Et_2Zn: $C_4H_{10}Zn$, M_r = 123.49, T = 100 K, tetragonal space group $I4_1$md, a = 9.893(7), c = 5.300(4) Å, V = 518.8(6) Å^3, Z = 4, ρ = 1.581 g cm^{-3}, $2\theta_{max}$ = 50°, 238 unique reflections (coverage 0.93), R_{int} = 0.0303, $R1$ ($I > 2\sigma(I)$) = 0.0286, $wR2$ (all data) = 0.0694. Zn and C-atoms were refined anisotropically, while H-atoms were constrained to parent C-atoms.

[S1] G. M. Sheldrick, *Acta Crystallogr.* **2008**, *A64*, 112.

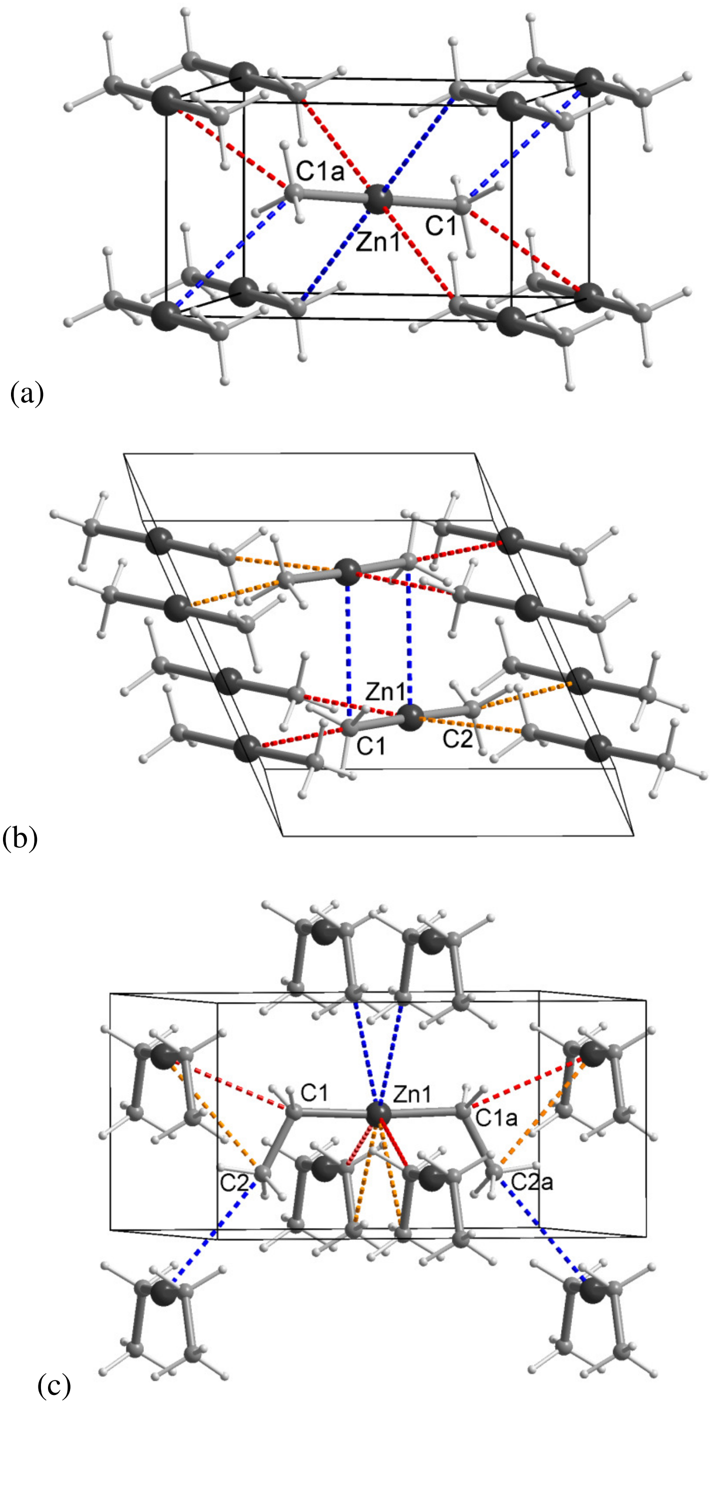

Figure S1: Shortest intermolecular Zn...C distances. α-Me_2Zn (a): 3.491(7) Å (red), 3.708(7) Å (blue); β-Me_2Zn (b) 3.327(14) Å (red), 3.424(14) Å (orange), 3.455(15) Å (blue); Et_2Zn (c) 3.254(6) Å (red), 3.482(6) Å (orange), 3.504(6) Å (blue).

2. Structural comparison of α-Me_2Zn and β-Me_2Zn

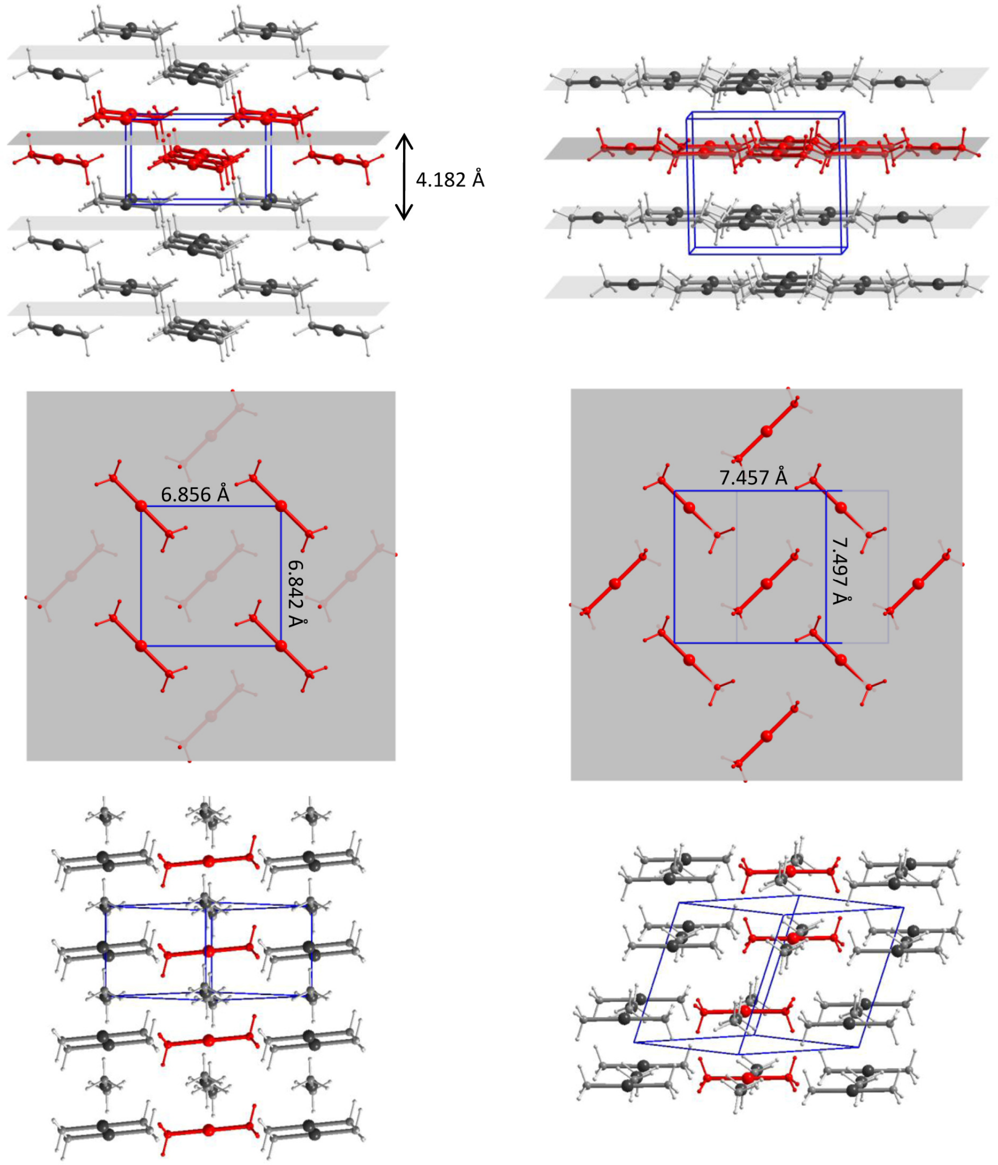

Figure S2: Crystal packing diagrams of α-Me_2Zn (left) and β-Me_2Zn (right), (a) view along the *a*-axis, (b) view onto the *ab* plane, (c) view along [110]. In (a) and (b) the mean planes through the layers of molecules are shown in gray and one layer is highlighted in red. (c) highlights the stacking of molecules along *c*; such stacks are shown in red. β-Me_2Zn forms compact layers parallel to the *ab*-plane, which are more or less planar. These layers contain molecules aligned in two different orientations perpendicular to each other forming T-shaped interactions which yield a square grid type of layer arrangement. The structure of α-Me_2Zn also features molecules of two orientations that are perpendicular to each other, but these are arranged in alternate layers. Thus a set of two neighboring layers (shown in red in (a)) can be regarded as a corrugated analogue of the planar layer of β-Me_2Zn. Compared to β-Me_2Zn, the structure of α-Me_2Zn is compressed in *a* and *b* direction (cell parameters *a* and *b* decrease

from around 7.5 Å in β-Me_2Zn to around 6.8 Å in α-Me_2Zn, while on the other hand the distance between layer planes increases from 3.4 Å (β-Me_2Zn) to 4.2 Å (α-Me_2Zn). Hence, the structures of both phases can be compared as follows: While β-Me_2Zn forms more compact layers in the form of a planar square grid in the *ab*-plane, these layers are stacked in lower symmetric fashion along the *c*-direction. In contrast, molecules of α-Me_2Zn are aligned effectively in the form of linear stacks along *c* but show a less compact arrangement in both *a* and *b* directions.

3. Hirshfeld surface analysis

Hirshfeld surface analysis was carried out for the X-ray crystal structures of α-Me_2Zn (Fig. S3), β-Me_2Zn (Fig. S4) and Et_2Zn (Fig. S5) using the program CrystalExplorer (see ref. [S2] for a detailed description of the method).

The Hirshfeld surface partitions the crystal into molecular entities. Color coding the surface according to certain surface properties, such as distance to nearest atom and curvedness, provides a useful visual tool to examine the intermolecular interactions in crystals structures. Note that C-H bond lengths are normalized by CrystalExplorer to 1.083Å.

d_e,is the distance from the Hirshfeld surface to the nearest nucleus *outside* the surface, d_i, the distance to the nearest nucleus *inside* the surface. Red regions on the surface represent shorter, green intermediate and blue longer distances.

The curvedness is a function of the root-mean-square curvature of the surface, with flat areas of the surface having a low curvedness and areas of sharp curvature having a high curvedness. Areas on the Hirshfeld surface with high curvedness tend to divide the surface into contact patches with each neighboring molecule, so that the curvedness of the Hirshfeld surface could be used to define a coordination number in the crystal.

2D 'fingerprint' plot: Each point corresponds to a unique (d_e, d_i) pair. Points are colored blue for a small, green medium to red for points with the greatest contribution. These plots are pseudo-mirrored along the $d_e = d_i$ diagonal. Features along the diagonal occur due to H···H contacts, while the 'wings' are due to H···Zn interactions.

The surfaces of α- and β-Me_2Zn show very similar features: 85% of the molecular surface is taken up by hydrogen and 15% by zinc, while H…H contacts engage about ¾ and H…Zn contacts ¼ of the molecular surface. The Hirshfeld surfaces of both Me_2Zn phases show small contributions of direct Zn...Zn contacts (0.9 % for the α- and 0.6 % for the β-phase). However, the corresponding Zn…Zn distances of 4.183(3) (Me_2Zn) and 4.079(7) Å (β-Me_2Zn) are somewhat long suggesting the absence of significant interactive forces between the metal atoms. In Et_2Zn the surface contribution of Zn is reduced to about 10%, while Zn…H contacts make up 17 %. The shortest H…H interactions of β-Me_2Zn and Et_2Zn measure about 2.4 Å, which is equal to the sum of the Van-der-Waals radii. Slightly longer H…H contacts of 2.6 Å are observed in the more loosely packed α-phase of Me_2Zn. On the other hand, the shortest Zn…H contacts in all three structures are very similar (around 2.8 Å).

[S2] (a) J. J. McKinnon, D. Jayatilaka M. A. Spackman, *Chem. Commun.* **2007**, 3814.
(b) M. A. Spackman, D. Jayatilaka, *CrystEngComm* **2009**, *11*, 19.

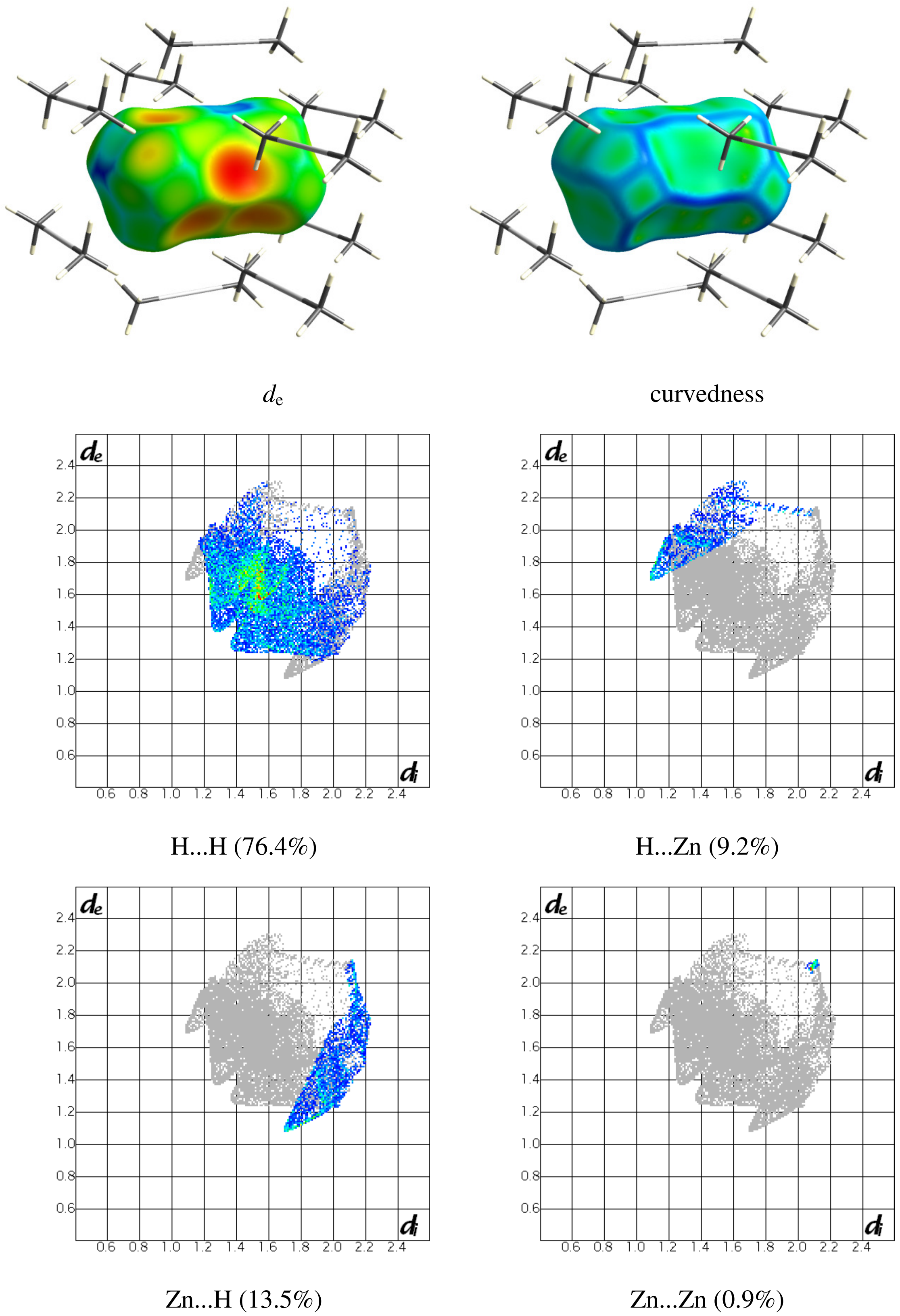

Figure S3: Hirshfeld surface and fingerprint plots of the $P2_1$/n structure of α-Me_2Zn. Counting the green areas on the surface depicting curvedness reveals that each molecule makes contact to 14 neighbors.

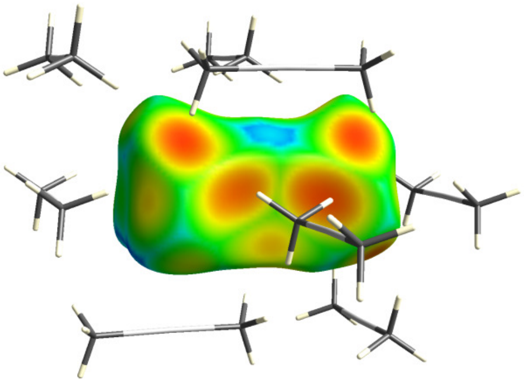

d_e

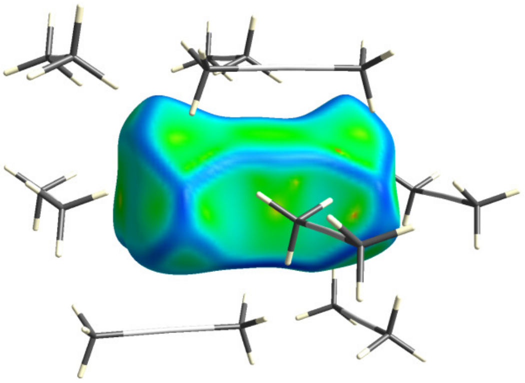

curvedness

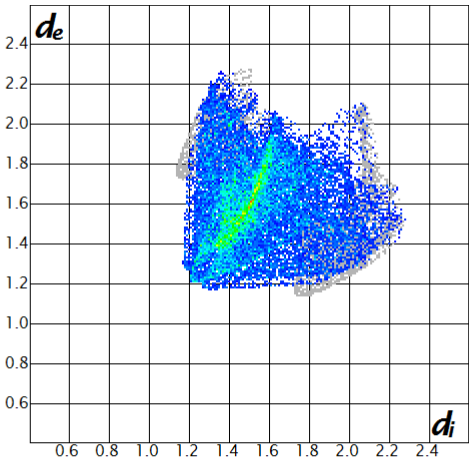

H...H (75.7%)

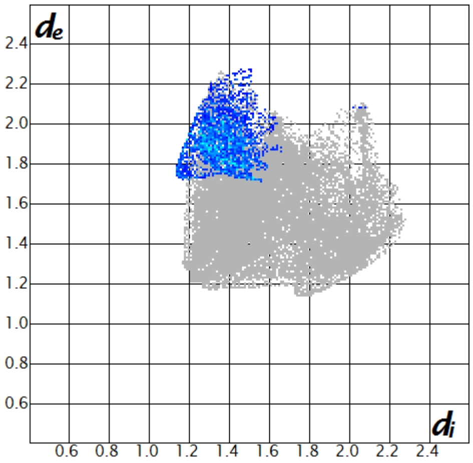

H...Zn (10.0%)

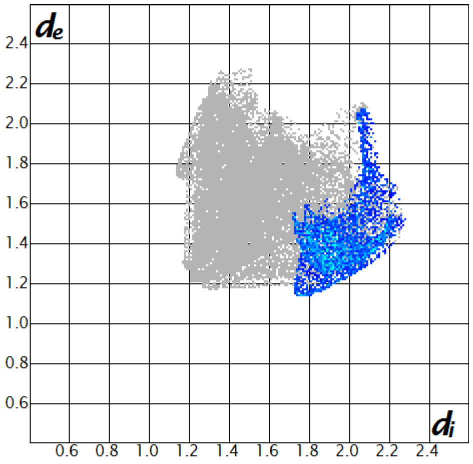

Zn...H (13.7%)

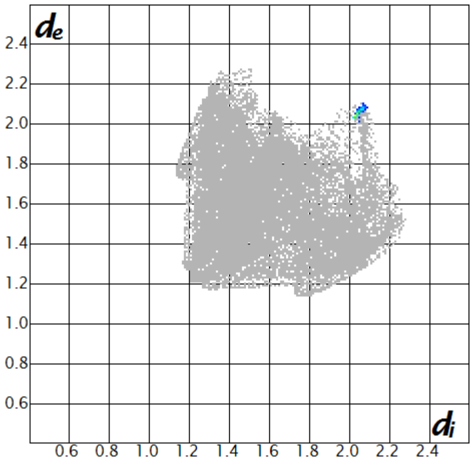

Zn...Zn (0.6%)

Figure S4: Hirshfeld surface and fingerprint plots of the crystal structure of β-Me_2Zn. Similar to α-Me_2Zn, each molecule in β-Me_2Zn makes direct contact to 14 neighbors.

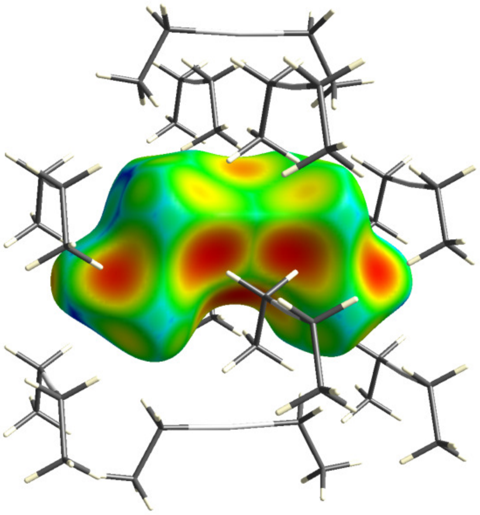

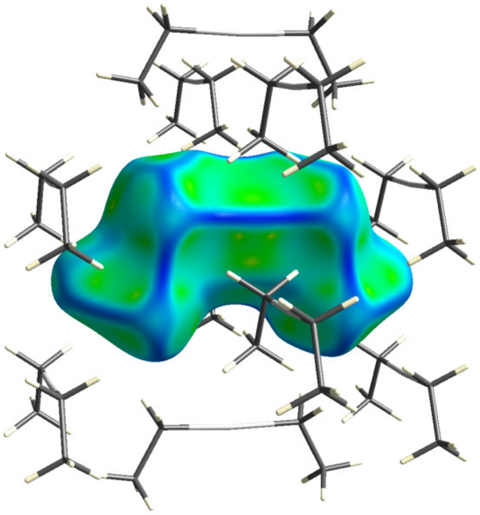

d_e curvedness

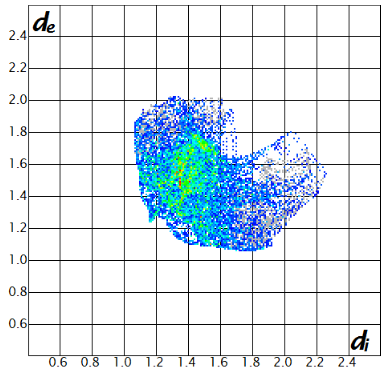

H...H (82.8%)

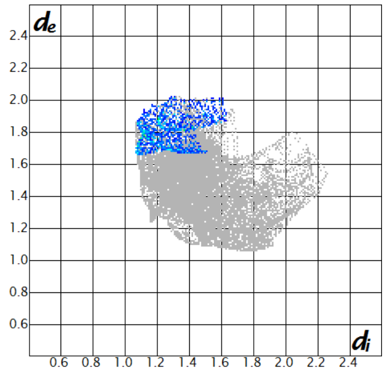

H...Zn (7.3%)

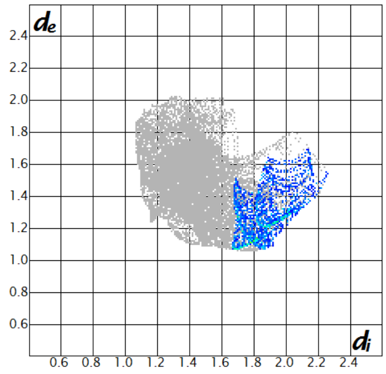

Zn...H (9.9%)

Figure S5: Hirshfeld surface and fingerprint plots of structure of Et_2Zn.

4. Two-dimensional disorder of α-Me_2Zn

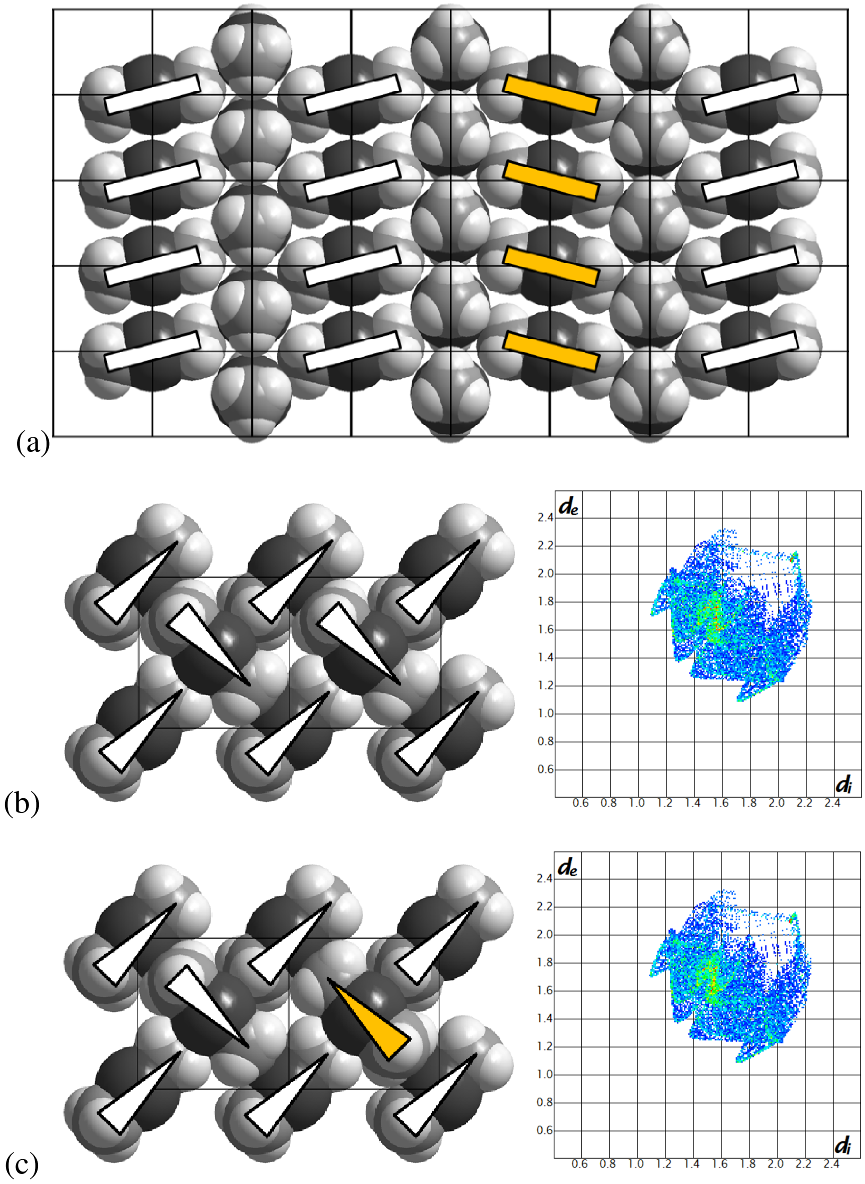

Figure S6: (a) Stacks of molecules along the *c*-axis can adopt two tilt-orientations (shown in white and orange) with respect to the horizontal *ab*-plane. (b) Fingerprints of Hirshfeld surfaces of the $P2_1$/n structure of α-Me_2Zn (viewed along *c*) and (c) the two-dimensionally disordered form. The similarity of fingerprint plots shows that the intermolecular interactions are virtually identical in both the ordered and disordered form. This suggests that crystals of α-Me_2Zn are likely to be affected by two-dimensional disorder in *a* and *b*, while one-dimensional order is maintained within the stacks of molecules along *c*. Diffuse scattering, which is indicative of this type of disorder, was too weak to be detected, possibly due to the *I*-centered superlattice of Zn atoms, which is not affected by the disorder but dominates the reflection pattern.

5. Assessment of the conformation of the methyl groups of β-Me_2Zn

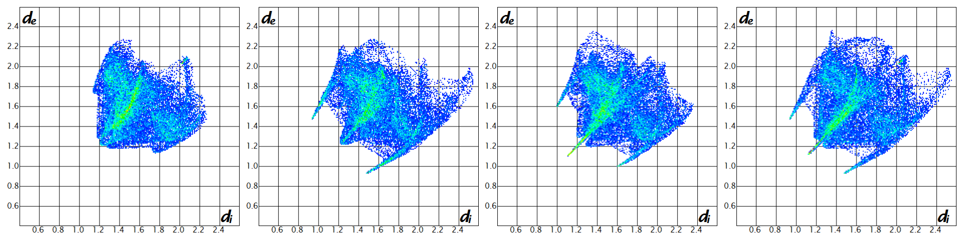

	A	B	C	D
rot(C1)	0°	60°	0°	60°
rot(C2)	0°	60°	60°	0°
conformation	eclipsed	eclipsed	staggered	staggered
H...H (Å)	2.4	2.4	**2.2**	**2.2**
Zn...H (Å)	2.8	**2.4**	**2.6**	**2.4**

Figure S7: Hirshfeld surface analysis was used to determine the most appropriate orientation for the methyl groups in the structure of β-Me_2Zn. Methyl groups were rotated and their Hirshfeld surfaces assessed at various stages. The conformer **A** shows the most appropriate intermolecular distances, which are very similar to those observed in α-Me_2Zn and Et_2Zn. All other conformers (such as **B**, **C** and **D**) generate excessively short intermolecular contacts as indicated by the spikes in the fingerprint plots (excessively short contacts are underlined in the table above). Central spikes are due to short H…H contacts and those at the wings due to short Zn…H contacts. Also note that these structures tend to generate fringes in the region of longer contacts, which is symptomatic of void space.

6. Details of Density Functional Calculations

Initial calculations were performed with the plane-wave basis set VASP[S3] code using the Projector Augmented Wave method[S4] and the PBE functional.[S5] k-point sets were varied from 3x3x4 to 5x5x8 with little effect. A plane-wave cutoff energy of 600 eV was used with geometry optimization continued until the forces per atom were either 0.01 eV/Å or 0.005 eV/Å (both yielded essentially similar results). In the absence of van der Waals corrections, this approach unsurprisingly leads to overestimation of the lattice constants by 5% for *a* and 10% for *c* for α-Me_2Zn, with the overstimation of ~4 % for Et_2Zn. Using VASP version 5.2.11, calculations were also run with van der Waals corrections included with the Grimme DFT-D[S6] approximation. This substantially overbinds the solid causing contractions of 7% and 11% for *a* and *c* respectively.

Subsequently all structures were relaxed using dispersion-corrected density functional theory with the PBE generalized gradient approximation[S5] and the TS'09 dispersion correction[S7] as implemented in all-electron code FHI-aims.[S8] We first relaxed the structure and unit cell using a tier1 basis set (e.g. double-numeric plus polarization),[S8] followed by a highly converged relaxation of the atoms only using a tier2 basis set.[S8] In each case, the calculation was continued until the forces on each atom or the energy gradient with respect to each lattice vector coordinate was less than 0.01 eV/Å. The Brillouin zone was sampled with a 3x3x4 Monkhorst-Pack[S9] k-point grid for relaxing the high-T structure of Me_2Zn and a 3x3x2 grid for the other unit cells. This gave the best agreement with experiment for the lattice constants of α -Me_2Zn, with errors of 0.9% and 3.4% for *a* and *c* respectively. To obtain the projected density of states, a much denser grid of 16x16x24 k-points was used together with a Mulliken analysis. Finally, an uncertainty analysis[S10] was performed on the van der Waals contribution to the binding energy, which provides some measure of the absolute accuracy of the methods for this particular system.

The final structure obtained with FHI-aims for α -Me_2Zn was used as the start point for optimization with the van der Waals density functional (optB86b-vdwDF) as implemented in VASP.[S11] With a cutoff energy of 600 eV and a 3x3x5 k-point set the lattice constants reduce slightly to *a* = 6.74 Å (6.79 Å from FHI-aims) and *c* = 3.93 Å (4.04 from FHI-aims).

[S3] G. Kresse, J. Furthmüller, *Phys. Rev. B* **1996**, *54*, 11169.

[S4] P. E. Blöchl, *Phys. Rev. B* 1994, *50*, 17953, G. Kresse, D. Joubert, *Phys. Rev. B* **1999**, 49, 1758.

[S5] J. P. Perdew, K. Burke, M. Ernzerhof, *Phys. Rev. Lett.* 1996, *77*, 3865.

[S6] S. Grimme, *J. Comp. Chem.* **2006**, *27*, 1781.

[S7] A. Tkatchenko, M. Scheffler, *Phys. Rev. Lett.* **2009**, *102*, 073005.

[S8] B. Blum, R. Gehrke, F. Hanke, P. Havu, V. Havu, X. Ren, K. Reuter, M. Scheffler, *Comput. Phys. Commun.* **2009**, *180*, 2175.

[S9] H. J. Monkhorst, J. D. Pack, *Phys. Rev. B* **1976**, *13*, 5188.

[S10] F. Hanke, *J. Comp. Chem.* **2011**, *32*, 1424.

[S11] J. Klimes, D. R. Bowler, A. Michaelides, *Phys. Rev. B* **2011**, *83*, 195131.

7. Atomic coordinates for all calculated crystal structures

All coordinates are given in the geometry input format for FHI-aims, which contains the Cartesian lattice vector coordinates in lines labeled ‘lattice_vector’ and the Cartesian atomic coordinates followed by the type of atom in lines labeled ‘atom’. The unit cells are primitive cells in each case. Lines marked ‘#’ are comments and introduce each structure. Notice that the number of digits quoted for each coordinate merely serves to reproduce the exact input files and does not represent a measure of absolute precision for these coordinates.

```
# alpha Me2Zn
# Cohesive energy  = 0.609(41) eV/molecule, of which 93.9% is due to van der Waals forces.
lattice_vector 6.7726622000000001 0.0000000000000000 0.0000000000000000
lattice_vector 0.0000000000000000 6.8063748999999998 0.0000000000000000
lattice_vector 0.0000000000000000 0.0000000000000000 4.0364271000000000
atom    3.3870604700000002 3.4025049599999999 2.0177240099999998     Zn
atom    0.0002863500000000 -0.0002856100000000 -0.0000519100000000   Zn
atom    -1.3313152800000001 1.3649720000000001 -0.3159321200000000   C
atom    1.3319362299999999 -1.3654717700000001 0.3160007000000000    C
atom    2.0591274600000000 2.0336432200000001 1.7017513399999999     C
atom    4.7143303000000003 4.7718462300000004 2.3342905699999998     C
atom    2.3624704900000002 1.0774380800000001 2.1483728499999999     H
atom    1.0883686699999999 2.3361177400000002 2.1168015200000001     H
atom    4.4105177600000003 5.7278889399999997 1.8876052600000000     H
atom    4.8385695499999999 4.9208424500000003 3.4154157500000002     H
atom    5.6856314000000001 4.4702870900000002 1.9199990400000000     H
atom    1.9337830700000000 1.8845549900000000 0.6207569700000000     H
atom    1.0296441900000000 -2.3242518599999999 -0.1258307500000000   H
atom    2.3005093900000002 -1.0620783199999999 -0.1033354600000000   H
atom    -1.0286382800000000 2.3238119300000002 0.1254850200000000    H
atom    -1.4615951100000000 1.5088059800000000 -1.3970475000000000   H
atom    -2.2997052800000000 1.0618166200000001 0.1040034200000000    H
atom    1.4616503100000000 -1.5095681200000000 1.3971541900000000    H
```

beta-Me2Zn, local minimum 1
Cohesive energy = 0.580(34) eV/molecule, of which 97.9% is due to van der Waals forces.
lattice_vector 6.4182385799999997 0.0000000000000000 -2.7692586800000001
lattice_vector 0.0000000000000000 7.8912432700000004 0.0000000000000000
lattice_vector 0.0000000000000000 0.0000000000000000 7.3081631500000004
atom 3.1593294200000002 1.8828838500000000 2.3627350300000001 Zn
atom 2.9844448899999998 4.1126985600000001 -1.7279766900000000 Zn
atom -0.0252910200000000 0.1543010700000000 0.0884364100000000 Zn
atom -0.2502376300000000 -2.0513031399999999 3.3189007699999999 Zn
atom 1.6939557400000000 2.8342299500000001 -1.0603268600000000 C
atom 4.2515396000000001 5.4061632299999998 -2.4070966800000000 C
atom 1.2705584700000001 -1.1138840600000000 -0.5897576700000000 C
atom -1.2950389099999999 1.4442698100000000 0.7688687900000000 C
atom 4.4642822799999999 3.1482032499999999 1.6981303100000000 C
atom 1.8806649000000000 0.6027720200000000 3.0457519799999999 C
atom -1.5587430000000000 -0.7951167600000000 3.9954836299999998 C
atom 1.0273835099999999 -3.3295593299999999 2.6305910400000001 C
atom 0.8592091800000000 3.3432658100000001 -0.5627266900000000 H
atom 1.2893918400000000 2.2161662899999999 -1.8723414300000001 H
atom 2.1781708800000001 2.1657658299999998 -0.3339155100000000 H
atom 5.2038045999999998 4.9440576199999997 -2.6950081199999998 H
atom 4.4490604899999999 6.1620278400000004 -1.6335296100000001 H
atom 3.8391011900000001 5.9278994799999998 -3.2811604700000001 H
atom 2.0883624699999999 -0.5962584000000000 -1.1062031699999999 H
atom 1.6989960700000000 -1.7194866900000001 0.2195449400000000 H
atom 0.7824452500000000 -1.7942764899999999 -1.3024448099999999 H
atom -2.2724090399999999 0.9922221400000000 0.9786556500000000 H
atom -1.4297139400000001 2.2449797000000000 0.0280002500000000 H
atom -0.9206179100000000 1.9063672700000001 1.6921485999999999 H
atom 5.3177105100000004 2.6300350099999998 1.2433393399999999 H
atom 4.8387052600000002 3.7926778500000000 2.5041641000000001 H
atom 4.0009801100000004 3.7912846299999998 0.9360106200000000 H
atom 0.9302632199999999 1.0726490700000000 3.3272305700000002 H
atom 1.6809410400000000 -0.1579605900000000 2.2775964399999999 H
atom 2.2874615299999999 0.0853576400000000 3.9249919100000001 H
atom -2.3974734999999998 -1.3212677700000000 4.4680698999999997 H
atom -1.9551400000000001 -0.1608957600000000 3.1916285900000001 H
atom -1.0903887600000000 -0.1417377500000000 4.7457660700000002 H
atom 2.0002377500000001 -2.8701353900000002 2.4157640900000001 H
atom 1.1727218500000001 -4.1315910300000001 3.3679563600000000 H
atom 0.6504539800000000 -3.7915961100000000 1.7082752900000000 H

beta-Me2Zn, local minimum 2
Cohesive energy = 0.598(37) eV/molecule, of which 96.6% is due to van der Waals forces.
lattice_vector 6.6668402599999999 0.0000000000000000 -3.2333400000000001
lattice_vector 0.0000000000000000 7.6170829400000004 0.0000000000000000
lattice_vector 0.0000000000000000 0.0000000000000000 7.2631722400000003
atom 0.1604116000000000 0.0358339900000000 0.1827676000000000 Zn
atom 3.4702328599999999 2.0802192599999998 2.2016695600000000 Zn
atom -0.2495740600000000 5.8906512099999997 3.4353028200000000 Zn
atom 3.1120468799999998 3.8443157499999998 -1.8096944600000000 Zn
atom -1.4175895800000000 7.2015943599999996 4.2460669800000002 C
atom 0.9277534300000000 4.5891828800000001 2.6274336599999999 C
atom 4.6474856600000001 3.3796214800000000 1.3851054800000000 C
atom 2.2867728100000000 0.7889013800000000 3.0171100900000001 C
atom 1.3299627599999999 -1.2775968700000000 -0.6217449000000000 C
atom -1.0241609199999999 1.3344906900000000 0.9846292000000000 C
atom 1.9520048400000001 2.5303532700000000 -0.9919017900000000 C
atom 4.2830876199999999 5.1422473300000000 -2.6324175599999999 C
atom -2.3028684699999999 7.3889766400000001 3.6228059799999999 H
atom -0.8791397100000000 8.1504838900000003 4.3743299499999999 H
atom -1.7577498700000000 6.8447396200000004 5.2283297500000003 H
atom 0.4728587100000000 4.1565130999999997 1.7273510000000001 H
atom 1.1038767500000000 3.7699646000000002 3.3387740699999999 H
atom 1.8940247100000001 5.0280453800000000 2.3513701100000000 H
atom 5.5357947200000002 3.5619083400000000 2.0054904300000000 H
atom 4.1174171499999996 4.3327647300000001 1.2527337400000000 H
atom 4.9821500399999996 3.0154512499999999 0.4036140400000000 H
atom 2.7389161299999998 0.3616845200000000 3.9211597399999998 H
atom 2.1059606700000000 -0.0355964000000000 2.3129360100000000 H
atom 1.3223868400000001 1.2347546199999999 3.2884477699999999 H
atom 2.2066148200000000 -1.4746655399999999 0.0106730800000000 H
atom 0.7864420900000000 -2.2221001700000000 -0.7613083899999999 H
atom 1.6832681899999999 -0.9176709500000000 -1.5981183000000001 H
atom -0.5721407100000000 1.7764740900000000 1.8815585500000001 H
atom -1.2082164799999999 2.1481949999999999 0.2688782900000000 H
atom -1.9864344599999999 0.8890252500000000 1.2640427200000000 H
atom 1.0795129999999999 2.3167506200000001 -1.6245798100000000 H
atom 2.5043344099999998 1.5930093000000001 -0.8387651000000000 H
atom 1.5929119500000000 2.8984505800000000 -0.0206670400000000 H
atom 3.8193995400000000 5.5727341900000003 -3.5290721199999999 H
atom 4.4705627000000003 5.9638288399999997 -1.9266413099999999 H
atom 5.2451583499999996 4.7000383200000000 -2.9175691900000000 H

beta-Me2Zn, local minimum 3
Cohesive energy = 0.598(37) eV/molecule, of which 96.6% is due to van der Waals forces.
lattice_vector 6.5502371699999999 0.0000000000000000 -3.2719354900000002
lattice_vector 0.0000000000000000 7.6818903699999996 0.0000000000000000
lattice_vector 0.0000000000000000 0.0000000000000000 7.3331755100000002
atom 0.1440710200000000 0.0555186900000000 0.1955230400000000 Zn
atom 3.4184458800000002 2.0937385700000002 2.2265752700000001 Zn
atom -0.2304451100000000 5.9280040700000001 3.4445226400000002 Zn
atom 3.0411593500000000 3.8865796000000001 -1.8514331100000001 Zn
atom -1.3884281900000000 7.2395826400000001 4.2675511400000001 C
atom 0.9428214100000000 4.6296231499999996 2.6264337200000001 C
atom 4.5725503999999999 3.3967146300000000 1.3838046799999999 C
atom 2.2613447299999998 0.7977931500000000 3.0721638499999999 C
atom 1.2978377299999999 -1.2574248299999999 -0.6313390500000000 C
atom -1.0237394000000000 1.3525243899999999 1.0236979500000001 C
atom 1.8722501700000000 2.5944354500000002 -1.0124726799999999 C
atom 4.2194484299999999 5.1695124899999998 -2.6876233100000002 C
atom -2.2546806099999999 7.4695252099999996 3.6323307100000002 H
atom -0.8279210000000000 8.1694609200000006 4.4356610200000004 H
atom -1.7564244800000000 6.8621904999999996 5.2316245700000001 H
atom 1.1512886600000001 3.8247162399999999 3.3458120899999999 H
atom 1.8949690699999999 5.0773644899999999 2.3180800000000001 H
atom 0.4703588500000000 4.1758559200000001 1.7460643800000000 H
atom 5.4414341799999999 3.6302084699999999 2.0141816200000000 H
atom 4.0137313800000003 4.3264047699999999 1.2088080800000001 H
atom 4.9368453700000003 3.0094793200000001 0.4222552400000000 H
atom 2.0476255299999999 -0.0169284800000000 2.3655498800000001 H
atom 1.3110030900000000 1.2426434600000000 3.3902774600000001 H
atom 2.7493618500000001 0.3570168500000000 3.9507326900000002 H
atom 1.6732849299999999 -0.8784751900000000 -1.5919620500000000 H
atom 2.1589057299999999 -1.4962594800000000 0.0076680500000000 H
atom 0.7313194500000000 -2.1824649900000002 -0.8058820300000000 H
atom -0.5368841300000000 1.8183361300000001 1.8898786800000000 H
atom -1.2523402200000000 2.1492185600000000 0.3013415100000000 H
atom -1.9664212000000001 0.9016125500000000 1.3557233500000001 H
atom 1.0024296900000000 2.3675859899999998 -1.6439804000000000 H
atom 2.4225071699999998 1.6600731300000000 -0.8349492300000000 H
atom 1.5095498500000000 2.9861511400000000 -0.0521768800000000 H
atom 4.4309372900000001 5.9841015500000001 -1.9800733100000001 H
atom 5.1699874899999996 4.7138296100000003 -2.9894751799999999 H
atom 3.7493530499999999 5.6125278400000003 -3.5746877700000002 H

beta-Me2Zn, local minimum 4

Cohesive energy = 0.593(36) eV/molecule, of which 94.8% is due to van der Waals forces.

lattice_vector 6.9751289999999999 0.0000000000000000 -2.9974112700000002
lattice_vector 0.0000000000000000 7.5110095299999999 0.0000000000000000
lattice_vector 0.0000000000000000 0.0000000000000000 7.1487619799999997
atom 0.1418297500000000 0.8420462500000000 1.9381038200000000 Zn
atom 3.6791850500000001 2.9231590199999999 3.9994756800000002 Zn
atom -0.1418298200000000 6.6689631699999996 5.2106582699999997 Zn
atom 3.2959439599999998 4.5878505900000004 0.1518748300000000 Zn
atom -1.3148675100000000 8.0384014500000003 5.9114316300000000 C
atom 1.0444040500000000 5.3075986300000002 4.5253104300000002 C
atom 4.8494610800000002 4.2912438300000000 3.2900041500000001 C
atom 2.4956754299999999 1.5627518600000001 4.6925663899999996 C
atom 1.3148675000000001 -0.5273919100000000 1.2373303000000000 C
atom -1.0444040699999999 2.2034108899999998 2.6234515100000002 C
atom 2.1256679599999999 3.2197658400000000 0.8613464800000000 C
atom 4.4794535299999998 5.9482577900000004 -0.5412158000000000 C
atom -2.1714402499999998 8.2095099900000008 5.2445835599999997 H
atom -0.7736567300000000 8.9877164100000009 6.0253063400000002 H
atom -1.7040462100000000 7.7282086799999998 6.8920984900000004 H
atom 1.1241815900000001 4.4924165900000004 5.2578650500000004 H
atom 0.6650928900000000 4.8790341599999998 3.5886461399999998 H
atom 2.0462223599999998 5.7146063299999996 4.3392662700000004 H
atom 5.7018385199999999 4.4711867600000001 3.9602274099999999 H
atom 4.3057946100000004 5.2379495699999996 3.1664107300000000 H
atom 5.2443727200000003 3.9772152200000002 2.3130753799999999 H
atom 2.4526532799999998 0.7235633100000000 3.9847745300000001 H
atom 2.8536388600000002 1.1729812100000001 5.6542485800000000 H
atom 1.4815822800000000 1.9575220700000000 4.8342784500000002 H
atom 2.1714402800000001 -0.6985004800000000 1.9041783100000000 H
atom 0.7736567500000000 -1.4767068800000001 1.1234555399999999 H
atom 1.7040461499999999 -0.2171990200000000 0.2566634600000000 H
atom -1.1241815300000000 3.0185928799999999 1.8908968100000001 H
atom -0.6650929500000000 2.6319754400000002 3.5601157699999999 H
atom -2.0462224199999999 1.7964032500000000 2.8094956199999999 H
atom 1.2732905000000001 3.0398228100000000 0.1911232900000000 H
atom 2.6693344600000000 2.2730601199999998 0.9849399400000000 H
atom 1.7307562800000000 3.5337944200000000 1.8382752400000000 H
atom 4.5224757499999999 6.7874463199999999 0.1665760900000000 H
atom 4.1214900999999999 6.3380285100000000 -1.5028979499999999 H
atom 5.4935466699999997 5.5534875399999999 -0.6829278800000000 H

Et2Zn

Cohesive energy = 0.885(57) eV/molecule, of which 99.1% is due to van der Waals forces.

lattice_vector 4.6232100899999997 -5.8803702900000001 -0.9049191900000000
lattice_vector 0.0000000000000000 7.5224201600000002 -0.8515562800000001
lattice_vector 0.0000000000000000 0.0000000000000000 7.5985618900000000

atom	0.0300504100000000	0.0232315500000000	-0.0046903800000000	Zn
atom	3.4949125900000002	-2.5162603400000001	2.9013833199999999	Zn
atom	3.5176184600000000	-1.0367313300000001	4.1742429699999999	C
atom	2.3122152499999999	-0.9350708300000000	5.1138872600000003	C
atom	0.9519147000000000	-1.1264136100000000	1.2725092899999999	C
atom	0.0613697100000000	-1.9567897400000001	2.2019551499999999	C
atom	-0.8413423900000000	1.1818332100000000	-1.3089722300000000	C
atom	-2.3730611700000002	1.1777892299999999	-1.3019794600000001	C
atom	3.5310359999999998	-3.9776753199999999	1.6080677100000000	C
atom	2.3098262799999998	-4.9026152600000001	1.5866502199999999	C
atom	4.4492488100000003	-1.1355132200000000	4.7535227899999999	H
atom	3.6232962099999999	-0.1135039300000000	3.5830295900000002	H
atom	2.3931035600000001	-0.0814380200000000	5.8116759000000000	H
atom	2.1939171499999999	-1.8324821600000001	5.7343804399999998	H
atom	1.3707417500000001	-0.8000489400000000	4.5659523899999996	H
atom	1.6126786399999999	-1.7823663100000000	0.6840193100000000	H
atom	0.6483259700000000	-2.5753779200000002	2.9055007200000000	H
atom	-0.5855856300000000	-2.6478503400000002	1.6464203799999999	H
atom	-0.4603682600000000	0.8784939400000000	-2.2967524899999998	H
atom	-2.7993117000000001	1.8493473700000000	-2.0698706200000001	H
atom	-2.7851168899999998	0.1799787100000000	-1.4991810500000000	H
atom	3.6809553300000002	-3.5152184300000000	0.6195152400000000	H
atom	2.4015553199999999	-5.7033267199999997	0.8300403300000000	H
atom	1.3841980500000000	-4.3613105200000000	1.3533249400000000	H
atom	-0.4573565400000000	2.1990110700000001	-1.1324283399999999	H
atom	-2.7835998100000001	1.5082455200000000	-0.3393554100000000	H
atom	4.4460199200000003	-4.5545017799999998	1.8165497600000000	H
atom	2.1519468300000000	-5.4032953199999998	2.5505362400000000	H
atom	1.6153059700000001	-0.4710425800000000	1.8586492699999999	H
atom	-0.5959819600000000	-1.3286870799999999	2.8164370900000000	H
